# Phase Evolution During High-Energy Ball Milling and Annealing of Ti-Doped Mo-V-Si-B Alloys

**DOI:** 10.3390/ma18112494

**Published:** 2025-05-26

**Authors:** Dennis Zang, Julia Becker, Ulf Betke, Georg Hasemann, Kateryna Khanchych, Bronislava Gorr, Manja Krüger

**Affiliations:** 1 Institute of Materials, Technologies and Mechanics (IWTM), Otto-von-Guericke University Magdeburg, Universitätsplatz 2, 39106 Magdeburg, Germany; 2 Institute for Applied Materials (IAM), Karlsruhe Institute of Technology (KIT), Kaiserstraße 12, 76131 Karlsruhe, Germany

**Keywords:** Mo-Si-B, mechanical alloying, lattice parameter, microstructure, heat treatment

## Abstract

Refractory metal-based Mo-Si-B alloys have long been considered the most promising candidates for replacing nickel-based superalloys in the aerospace and energy sector due to their outstanding mechanical properties and good oxidation of the Mo-silicide phases. In general, the addition of vanadium to Mo-Si-B alloys leads to a significant density reduction, while small amounts of titanium provide additional strengthening without changing the phase evolution within the Mo_ss_-Mo_3_Si-Mo_5_SiB_2_ phase field. In this work, high-energy ball milling studies on Mo-40V-9Si-8B, substituting both molybdenum and vanadium with 2 and 5 at. % Ti in all constituents, were performed to evaluate the potential milling parameters and investigate the effects of Ti doping on the milling characteristics and phase formation of these multicomponent alloys. After different milling durations, the powders were analysed with regard to their microstructure, particle size, oxygen concentration and microhardness. After heat treatment, the silicide phases (Mo,V)_3_Si and (Mo,V)_5_SiB_2_ precipitated homogeneously within a (Mo,V) solid solution matrix phase. Thermodynamic phase calculations using the CALPHAD method showed good agreement with the experimental phase compositions after annealing, confirming the stability of the observed microstructure.

## 1. Introduction

The potential for improving nickel-based superalloys, currently used in jet engines and power plants, is severely limited due to their comparatively low melting temperature [1]. Therefore, molybdenum–silicide alloys, which not only satisfy requirements like high melting temperature and acceptable density but also exhibit appropriate oxidation and creep resistance, have increasingly come into scientific and industrial focus during the last few decades [1,2]. Promising candidates for replacing nickel-based superalloys in the aerospace and energy sector are multiphase Mo-Si-B alloys, pioneered by Berczik [3,4].

It is well known that Mo-Si-B alloys with a continuous α-Mo matrix (Mo_ss_) and embedded, homogeneously distributed silicide phases (Mo_3_Si and Mo_5_SiB_2_) provide the best combination of mechanical properties and oxidation resistance [5,6,7,8]. Such a microstructure can be realized via mechanical alloying (MA) and subsequent sintering and/or different methods for powder compaction. Mechanical alloying is a solid-state process in which a very homogeneous and fine microstructure can be produced using high-energy ball milling, which might lead to non-equilibrium states [9]. The principle is essentially based on repeated fracture and cold-welding processes of the individual powder particles [10]. The progress of mechanical alloying of powders primarily depends on the chosen milling parameters, especially the milling duration and the rotational speed. Krüger et al. [8] showed that a treatment of at least 20 h in a planetary ball mill PM400 with 200 rpm is necessary for Mo-Si-B alloys to achieve the above-mentioned type of microstructure during subsequent heat treatment. Likewise, some characteristic powder parameters change with milling time, such as lattice parameter, microstrain, particle size and microhardness [8,11,12,13]. These parameters dictate the milling progress, and they have a significant impact on the microstructure of the powders and the consolidated bulk material.

One disadvantage of Mo-rich Mo-Si-B alloys is their high density [2]. An interesting approach for reducing alloy density is to add lighter elements compared to Mo like Al, Cr, Ti and V. However, it was shown that larger amounts of Al, Cr and Ti in Mo-Si-B alloys lead to a reduction in the Mo solid solution fraction [14,15,16]. Becker et al. [17] were able to show that the addition of 40 at. % V to powder metallurgically processed (PM) Mo-9Si-8B alloys leads to a density reduction of approximately 17%. This phenomenon is directly related to the high solubility of V in Mo and the respective Mo-silicides, while V has only a minor influence on the phase fractions of the Mo_ss_-Mo_3_Si-Mo_5_SiB_2_ phase field. Apart from this, the addition of V significantly affects the mechanical properties of Mo-Si-B alloys.

Becker et al. [18] obtained slightly higher hardness values after the addition of 5 at. % V to Mo, which was attributed to the relatively weak lattice distortion caused by the vanadium atoms. In addition, the PM alloy Mo-40V-9Si-8B exhibits comparatively high room-temperature fracture toughness values of approximately 13.3 MPa $\sqrt{m}$, although these alloys have significantly higher oxygen concentrations due to the oxygen-contaminated V raw powder and the high affinity of V for oxygen [17]. It must be noted that Mo tends to significantly embrittle when oxygen is dissolved in the crystal lattice, even at low concentrations of around 10 ppm [19]. Although the brittle silicide phases in Mo-40V-9Si-8B and Mo-9Si-8B are similarly distributed, the addition of V results in a higher fracture toughness, which is due to both the slightly increased volume fraction of the tough Mo_ss_ phase and its size [7,17]. Unfortunately, the addition of high V concentrations to Mo-Si-B alloys also has an impact on the high-temperature mechanical properties, leading to a reduction in high-temperature compressive strength and creep resistance, as shown in [17].

Another promising alloying element in Mo-Si-B alloys is Ti, which will also reduce alloy density and at the same time has the potential to additionally strengthen the material via solid solution strengthening and second-phase or particle strengthening. Mo-Si-B alloys with Ti additions above 20 at. % are already well studied [20,21,22], with high Ti concentrations leading to the formation of the (Mo,Ti)_5_Si_3_ phase instead of the Mo_3_Si phase.

However, there is less information about the influence of low Ti concentrations (up to 5 at. %) in these materials. Inoue et al. [23] found that doping Mo with 1.0 and 1.5 mass% Ti, which were carburized via two-step heat treatment, leads to an improvement in hardness as a result of solid solution hardening. A similar conclusion was drawn by Becker et al. [18], who showed that the hardness of arc-melted Mo-5Ti is significantly higher compared to pure Mo due to solid solution hardening. According to Fan et al. [24], small amounts of Ti are sufficient to increase the tensile strength of pure Mo, since Ti forms a solid solution with Mo in a large solubility range up to 100 at. %, while at lower temperatures, the BCC solid solution will transfer to an HCP solid solution at higher Ti concentrations according to the phase diagram shown in [25]. In this context, it was shown that the addition of TiH_2_ to Mo powders during mechanical alloying has a higher impact on the tensile strength than the addition of pure Ti.

These observations align with more recent studies which also highlight the beneficial role of low Ti concentrations in Mo-based alloys. Hu et al. [26] demonstrated that even small amounts of Ti (0.5 wt.%) can significantly refine the grain size in pure Mo, enhance densification during sintering, and reduce oxygen impurity levels through the in situ formation of fine TiO_2_ or Ti_8_O_15_ particles. These oxide particles also act as effective grain boundary pinners, contributing to improved mechanical properties. Similarly, Choi et al. [27] reported that Ti additions in Mo-Si-B alloys led to partial solid solution formation within the Mo matrix and promoted the formation of intergranular TiO_2_, which influences fracture behaviour. Despite a moderate decrease in toughness, titanium proved effective in refining the microstructure and reducing oxygen levels, underlining its functional relevance even at minor concentrations.

While elemental Ti has shown these beneficial effects, TiH_2_ is considered even more suitable for powder-based processing routes, as its brittle nature promotes homogeneous dispersion within the Mo matrix during milling. During subsequent heat treatment, the hydrogen volatilizes and a Mo-Ti solid solution forms. The addition of TiH_2_ accelerates solid solution formation, since the smaller particle size of this compound reduces the diffusion paths of the decomposed Ti [24]. In addition, the H_2_ of the decomposed TiH_2_ can react with oxygen dissolved in the alloy to form H_2_O, which is then evaporated in the atmosphere. This reaction can effectively lower the oxygen concentration in the alloy [28]. Simultaneously, the gettering of oxygen is achieved by Ti at temperatures higher than 700 °C, reducing the free oxygen concentration in the material by forming Ti oxides, and is no longer reactively available in this form [29].

While previous studies have extensively addressed the mechanical alloying of Mo-Si-B alloys with vanadium as a major alloying element [17] and others have focused on high Ti contents (>20 at. %) leading to the formation of (Mo,Ti)_5_Si_3_ phases [20,21,22], the effects of small Ti additions (≤5 at. %) in combination with V remain largely unexplored. This study therefore introduces a novel alloying approach by combining Ti and V via TiH_2_-assisted mechanical alloying, with particular emphasis on phase evolution, particle size behaviour and oxygen uptake. The results are benchmarked against undoped and solely V-doped reference systems to systematically evaluate the influence of minor Ti additions.

## 2. Material and Methods

Elemental powders of Mo, V, Si, B and TiH_2_ of 99.95%, 99.5%, 99.5%, 98% and 99.5% purity, respectively, were used to produce different Mo-V-Si-B(-Ti) alloys via a multi-step powder metallurgical process. The elemental powders had different average particle sizes of 4 µm (Mo), 38 µm (B) and 44 µm (V, Si, TiH_2_). Mechanical alloying was carried out in a planetary ball mill (Retsch^®^ PM 100, Retsch, Haan, Germany) with a powder-to-ball weight ratio of 1:13 and a rotational speed of 300 rpm. Weighing, mixing and powder removal (after 1, 2, 5, 10 and 20 h of milling) were performed in a glovebox under protective argon atmosphere in order to keep the oxygen concentration in the powders as low as possible. In this study, stainless steel vials and balls (d = 10 mm) were used. In the first step, a milling study for the Mo-40V-9Si-8B alloy was carried out. In the second step, 2 at. % and 5 at. % Ti was added to this alloy composition to vary either the Mo or V concentrations (Mo-40V-9Si-8B-2Ti, Mo-40V-9Si-8B-5Ti, Mo-38V-9Si-8B-2Ti and Mo-35V-9Si-8B-5Ti). The mechanically alloyed powders obtained from the five milling studies were then characterized as a function of milling time.

The powders, finally milled for 20 h, were subjected to heat treatment under vacuum at 1400 °C for 1 h using a tube furnace (HTM Reetz LORA 1700-80-330-1, HTM Reetz GmbH, Berlin, Germany) and characterized as described above. Field-assisted sintering (FAST) of the mechanically alloyed powders was carried out in a hot press HP D (FCT System GmbH, Frankenblick, Germany) at the Karlsruher Institute of Technology (KIT). For the process, a heating rate of 100 K/min and a holding time of 15 min at 1100 °C and 1500 °C were chosen. The sintering process was performed under vacuum (<10 bar) and a uniaxial pressure of 50 MPa.

The quantitative phase analysis of the mechanically alloyed powders was performed with powder X-ray diffraction (XRD) using a D8 Discover X-ray diffractometer with Bragg–Brentano reflection geometry (Bruker-AXS GmbH, Karlsruhe, Germany). The diffractometer was equipped with an Eiger2 detector operating in 1D mode, as well as a 0.6 mm fixed divergence slit and 2.5° primary and secondary soller slits as optical components. The diffracted intensities were recorded between 10° and 160° 2θ using Co Kα_1,2_ radiation; Kβ radiation was removed via a 10 µm Fe foil placed in front of the detector opening. The obtained powder patterns were analysed by following the Rietveld technique using TOPAS V6 software (Coelho Software, Brisbane, Australia/Bruker-AXS) [30,31]. The Mo/V ratio in the identified BCC solid solution phases was determined from their respective unit cell volume under application of Vegard’s law [32]. A calibration curve was calculated from literature data of Mo, V and Mo-V solid solution phases obtained from the Crystallographic Open Database (COD) [33]. Besides the phase composition, the microstrain ε_0_ was determined for each phase from the reflection profiles. The contribution of the instrument to the reflection profiles was determined from a measurement of the NIST 660a standard (LaB_6_) using the Fundamental Parameter Approach [34,35]. The diffractograms were treated using the Fundamental Parameter Approach (FPA). An instrument function, describing the influence of the diffractometer on reflection shape, was determined from a reference measurement of the NIST 660a material (LaB_6_). This instrument function contained parameters describing the emission profile of the X-ray source, slits and axial divergence. Sample-related reflection broadening was treated using macros for size and strain broadening, where applicable.

Microstructures after different milling durations were characterized using an SEM (FEI ESEM XL30 FEG/Philips equipped with EDS, Hillsboro, OR, USA) and Zeiss EVO 15 (equipped with EDS, Carl Zeiss Microscopy GmbH (Jena, Germany) equipped with EDS, Oberkochen, Germany) in SE mode. For this purpose, the samples were first mounted in a cold embedding material (Technovit 4071, Heraeus Kulzer, Wehrheim, Germany) and then wet-ground (600, 1200, 2500 grit SiC paper) and polished (3 µm and 1 µm diamond suspension).

Measurements of particle size and particle size distribution were analysed using laser diffraction analysis (Mastersizer^®^ 2000, Malvern Panalytical, Malvern, UK) with water as the dispersion medium and evaluation of the results according to the Fraunhofer diffraction theory.

Determination of the oxygen concentration of the pure powders and the mechanically alloyed powders was achieved by using the ON/H-mat 286 analyser (G8 GALILEO, Bruker, Viersen, Germany).

Microhardness tests were conducted by using a Micro-Duromat 4000 E device (Reichert-Jung, Depew, NY, USA) on the embedded powder particles. For these tests, it is necessary to have a sufficient distance between the hardness indentation and the boundary of the particles in order to keep the influence of the embedding material on microhardness as low as possible. Since the particles are relatively small, the applied load was set to 0.1 N, which was applied for 10 s using a load rate of 0.01 N/s. The indentations were measured manually with ImageJ software (Version 1.54p), and the Vickers hardness value was calculated from the mean value of the two diagonals for a minimum of 15 indentations.

The mean centre-to-centre spacing between silicide phases was estimated using a simple stereological relation:

(1) $$\lambda =d\cdot {\left(\frac{1-f}{f}\right)}^{1/3}$$

where d is the average silicide width and f is the silicide volume fraction.

The theoretical density was calculated based on the phase fractions (ω*_i_*) and densities (ρ*_i_*) obtained from the Rietveld refinement. Subsequently, the overall density of the multi-phase material was determined using the inverse rule of mixtures, as follows:

(2) $$\rho_{theo}={\left(\sum_{i}\frac{\omega_{i}}{\rho_{i}}\right)}^{-1}$$

The density of the FAST alloys Mo-40V-9Si-8B and Mo-40V-9Si-8B-5Ti was measured by using Archimedes’ method with an analytical balance (Sartorius, Göttingen, Germany) at 20 °C, whereby each specimen was measured three times.

Using the CALPHAD (Calculation of Phase Diagram) method to develop the Mo-Si-B-V database, each phase of the system was modelled with a separate Gibbs energy expression as a function of temperature T and pressure p. These models contain parameters obtained by fitting experimental and theoretical data. A detailed description of the CALPHAD method is given in [36].

For a multicomponent system, thermodynamic data on its constituents (i.e., pure elements) as well as all of the subsystems included are required. In the present case, the quaternary Mo-Si-B-V system contains six binary and four ternary subsystems in addition to pure elements. Available experimental and theoretical literature data were utilized while modelling multicomponent phases. It has to be mentioned that neither experimental nor theoretical data for the two ternary subsystems Mo-Si-V and Mo-B-V are available in the literature. Taking this shortcoming into account, the database in its current state does not provide a full description, especially for the B- and Si-rich alloys within the Mo-Si-B-V system. Since Mo- and V-rich alloys are of primary interest in the present study, the thermodynamic modelling can be considered a first rough estimation of phase equilibria in the quaternary system. FactSage 8.2 software was used for thermodynamic calculations.

## 3. Results

### 3.1. X-Ray Diffraction (XRD) Measurements

**Figure 1:**
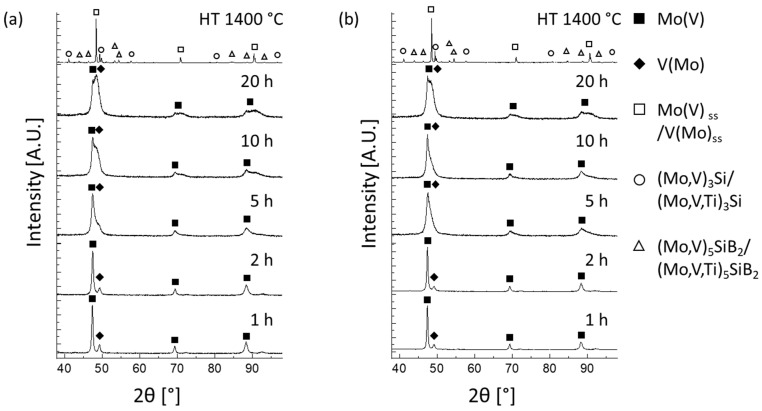
X-ray diffractograms of (**a**) Mo-40V-9Si-8B and (**b**) Mo-40V-9Si-8B-5Ti powders after different milling durations and after heat treatment at 1400 °C for 1 h, showing the progress of mechanical alloying and the phase evolution in the as-milled powders.

Since the diffractograms of the investigated alloys are largely similar, Figure 1 provides a representative example, displaying the X-ray diffractograms of the Mo-40V-9Si-8B and Mo-40V-9Si-8B-5Ti alloys at different milling times. As milling time increases, reflection broadening becomes more pronounced due to the high concentrations of V, Si and B, as well as Ti in the case of the Ti-alloyed powders, within the molybdenum lattice. Additionally, the reflections of molybdenum (approx. 47°) and vanadium (approx. 49°) in the lower 2θ range merge within the first hours of mechanical alloying, indicating the progressive dissolution of vanadium into the Mo solid solution (Mo_ss_). It is noteworthy that Mo and V can be entirely dissolved. However, the addition of small amounts of Ti to Mo-40V-9Si-8B does not noticeably affect the position of the reflections. It can be seen that a supersaturated Mo(V,Si,B) and Mo(V,Si,B,Ti) solid solution formed after 20 h of milling, where Si, B and Ti are dissolved quite quickly in the Mo solid solution during mechanical alloying. Diffraction patterns of the individual elements disappear even at the beginning of the mechanical alloying process, and the most significant Si pattern cannot be detected after 1 h of milling. These results are in agreement with those of Becker et al. [17] on similarly treated Mo-40V-9Si-8B.

Rietveld refinement reveals the existence of two solid solution phases for the milled samples: a V-rich V(Mo)_ss_ solid solution phase in which molybdenum is dissolved and a Mo-rich Mo(V)_ss_ solid solution phase in which V is dissolved. Table 1 presents a comparison of the evolution of the lattice parameter, microstrain (ε_0_) and phase fraction of the two solid solutions, with exemplary values shown for 2, 10 and 20 h of milling. With increasing milling time, the lattice parameter of Mo(V)_ss_ decreases, starting from a value of 3.147 Å for pure Mo, while the lattice parameter of V(Mo)_ss_ increases, starting from a value of 3.024 Å for pure V. During mechanical alloying, the values of the lattice parameters converge according to Table 1, but two solid solution phases can still be detected, even after milling for 20 h. The large difference in the lattice parameters of the Ti-doped powders is attributed to this effect, which means that the progress of solid solution formation is slower compared to the Ti-free alloys. This indicates that longer milling durations must be foreseen to entirely homogenize the Ti-containing powders.

The microstrain in V(Mo)_ss_ increases from approximately 3.5 × 10^−3^ after 2 h up to values ranging between 7 × 10^−3^ and 13.2 × 10^−3^ after 20 h of milling. In contrast, the microstrain in Mo(V)_ss_ is slightly higher at 2 h (around 5 × 10^−3^) but decreases after 20 h, ranging between 4.88 × 10^−3^ and 8.9 × 10^−3^. The increase in microstrain can be related to the high plastic deformations occurring during mechanical alloying due to the high-energy collision between milling balls and powders. Consequently, the dislocation density increases, leading to a higher degree of lattice defects in the microstructure [8,37,38].

The phase fraction of Mo(V)_ss_ initially increases but then decreases between 10 and 20 h of milling for all investigated alloys. On the other hand, the phase fraction of V(Mo)_ss_ continuously increases over the entire milling time. As expected, the phase fraction of pure Mo decreases, but after 20 h of milling there is still residual Mo left, which is not dissolved during the milling process, and therefore, a complete (Mo,V) solid solution has not yet formed. This incomplete dissolution, as evidenced by residual Mo reflections in the XRD pattern, may be attributed to the lower energy input of the PM100 planetary mill used in this study. Compared to the PM400 planetary mill used in prior studies [17], the PM100 mill employed here has a smaller sun wheel radius, which leads to reduced kinetic energy transfer during milling, despite the higher rotation speed. As the kinetic energy imparted to the powder is proportional to the square of the radius and rotation speed (E ∝ R^2^·ω^2^), the total energy input per unit powder mass is significantly lower in the present setup. This reduced energy input likely contributes to the incomplete dissolution of Mo observed after 20 h and indicates that either a higher energy milling setup or prolonged milling duration may be necessary for full alloying.

Previous investigations using the PM400 mill (Becker et al. [17]) reported complete Mo dissolution under similar milling durations. In the present study, the lower energy input of the PM100 mill likely limited the degree of Mo dissolution, despite the relatively long milling time of 20 h. Therefore, increasing the milling energy by using a higher rotational speed is expected to be more effective than merely extending the milling duration. Nevertheless, care must be taken to avoid negative side effects such as powder agglomeration, contamination or excessive work hardening, which may arise under high-energy milling conditions.

The concentration of V in Mo(V)_ss_ and Mo in V(Mo)_ss_ increases during milling, indicating the complete solubility of V in Mo and vice versa. The decrease in Mo concentration in the V(Mo)_ss_ phase between 10 and 20 h in Mo-40V-9Si-8B and Mo-35V-9Si-8B-5Ti, but not in the other alloys, suggests inhomogeneous milling progress in the mechanically alloyed powder batch. Initially, Mo dissolves into V(Mo)_ss_, but extended milling likely induces local supersaturation, promoting partial Mo segregation from the solid solution without distinct phase formation. In Mo-35V-9Si-8B-5Ti, the high Ti concentration may enhance Mo displacement from the V(Mo)_ss_ phase, while in the alloys with 2 at. % Ti, the solubility limit is not exceeded, preventing Mo depletion from V(Mo)_ss_. After 20 h, a steady state is reached, stabilizing the Mo concentration in the V-based solid solution. At this stage, the degrees of Mo and V dissolution in their respective solid solutions are nearly identical, except for the Mo-40V-9Si-8B alloy. This suggests that Mo and V dissolve in their respective lattice to a similar extent, which is more pronounced for the Ti-doped alloys.

After heat treatment of the mechanically alloyed powders at 1400 °C for 1 h under vacuum, the two silicide phases (Mo,V)_3_Si/(Mo,V,Ti)_3_Si (A15 structure) and (Mo,V)_5_SiB_2_/(Mo,V,Ti)_5_SiB_2_ (D8_l_ Structure) are formed and embedded in the (Mo,V)_ss_/(Mo,V,Ti)_ss_ matrix phase (BCC, A2 structure). It is important to note that the silicide phases (Mo,V)_3_Si and (Mo,V)_5_SiB_2_ were only observed after the annealing step and were therefore not included in Rietveld refinement of the as-milled powders. While the main reflections of the matrix and silicide phases are visible after heat treatment, some reflection overlap remains, particularly between the (Mo,V)_3_Si and (Mo,V)_5_SiB_2_ phases, due to their partially overlapping diffraction angles and similar scattering intensities. Nevertheless, both were included as separate phases in Rietveld refinement using well-established crystal structures. The formation of these silicides indicates that Si and B are primarily responsible for the precipitation of the A15 and D8_1_ phases during annealing, while V and Ti remain partially dissolved in all three phases. Finally, no additional phases were detected in Ti-doped samples, suggesting that Ti has no significant influence on the overall phase evolution during post-alloying heat treatment.

The lattice parameter of (Mo,V)_ss_ for the Mo-40V-9Si-8B alloy slightly increases after heat treatment compared to the as-milled state because some of the V and Si diffuse out of the (Mo,V)_ss_ to form the two silicide phases (Table 2). Vanadium (134 pm), being slightly smaller than Mo (139 pm), may reduce the lattice parameter upon diffusion, leading to a more compact atomic arrangement. In contrast, Si has a significantly smaller atomic radius (111 pm) and a strong tendency to form silicide phases, further depleting the solid solution and potentially causing additional contraction. However, since Mo is larger than both V and Si, its increased concentration in the solid solution is likely to result in moderate expansion of the lattice parameters of (Mo,V)_ss_. In the Ti-doped alloys, the lattice parameter slightly decreases due the slightly larger atomic radius of Ti (147 pm) compared to Mo, which consequently contributes to a higher lattice parameter of Mo(V)_ss_ after mechanical alloying. During heat treatment, some of the Ti diffuses from the supersaturated Mo(V)_ss_ and is dissolved in the respective silicide phases, causing a slight decrease in the lattice parameter.

However, the X-ray diffractograms in Figure 1 appear visually unchanged, as the reflex shift remains below the resolution limit of the XRD analysis. In the Mo-substituted alloys Mo-40V-9Si-8B-2Ti and Mo-40V-9Si-8B-5Ti, the phase fraction of the (Mo,V)_ss_ matrix phase slightly decreases with the addition of Ti, while the phase fraction of the two silicides increases. For the V-substituted alloys Mo-38V-9Si-8B-2Ti and Mo-35V-9Si-8B-5Ti, however, there is no significant change regarding the phase fractions compared to the Mo-40V-9Si-8B alloy.

**Figure 2:**
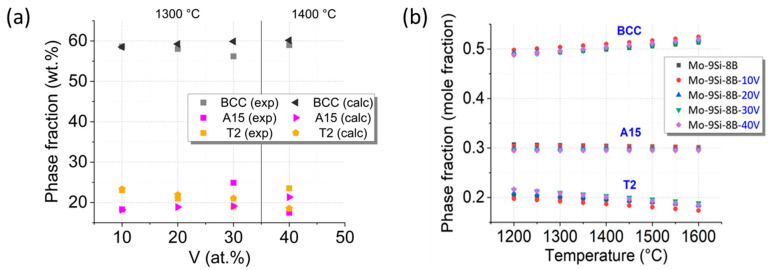
(**a**) The calculated (calc) and experimentally (exp) observed [17] equilibrium phase composition of Mo-xV-9Si-8B (x = 10, 20, 30 and 40 at. %) at 1400 °C. Note: experimental data for 10, 20 and 30 at. % V are taken from Becker et al. [17] and correspond to measurements at 1300 °C, while the 40 at. % V data were obtained in this study at 1400 °C. (**b**) The calculated equilibrium phase composition of Mo-xV-9Si-8B (x = 0, 10, 20, 30 and 40 at. %) in the temperature range 1200–1600 °C. The data for the V-free alloy Mo-9Si-8B comply with the thermodynamic assessment of the Mo-Si-B system presented in [39].

A comparison of the obtained experimental data with the theoretical results calculated using the self-developed thermodynamic database for a quaternary alloy system Mo-Si-B-V is performed. According to the theoretical assessment, the calculated equilibrium phase composition of the alloy Mo-40V-9Si-8B at 1400 °C yields 60.1, 21.3 and 18.5 wt.% for (Mo,V)_ss,_ (Mo,V)_3_Si and (Mo,V)_5_SiB_2_, respectively. These results are in good agreement with the experimental values given in Table 2. In order to further confirm the validity of the developed database in a wider concentration range of V, a comparative analysis of the calculated phase composition for Mo-xV-9Si-8B alloys with different V contents (x = 10, 20, 30 and 40 at. %) at 1400 °C and the experimental data [17] is performed (see Figure 2a). A very good agreement is obtained for 10 and 20 at. % V, while a slight deviation (up to 5 wt.%) is observed for 30 and 40 at. % V.

Figure 2b shows the calculated phase composition of the alloy Mo-xV-9Si-8B (x = 0, 10, 20, 30 and 40 at. %) in the temperature range 1200–1600 °C. Three-phase equilibria over the temperature range 1200–1600 °C are expected for all alloys analysed. The change in the phase composition with increasing temperature follows the same trends: the content of the BCC phase increases, the amount of the A15 phase ((Mo,V)_3_Si) changes not significantly, and, consequently, the amount of the T2 phase ((Mo,V)_5_SiB_2_) decreases. The essential implication of the diagram shown in Figure 2a is that the V additions—at least up to 40 at. %—do not notably alter the phase equilibria in the given temperature range. It can, therefore, be anticipated that the microstructure of the V-containing alloys resembles the microstructure of the Mo-9Si-8B counterpart, as proposed above. Moreover, the increasing temperature up to 1600 °C exhibits a negligibly small effect on the phase equilibria, suggesting a highly stable microstructure.

While the thermodynamic calculations presented in this study show good agreement with the experimental results, it must be emphasized that the available thermodynamic descriptions for the Mo-Si-B-V quaternary system are still limited due to the lack of comprehensive experimental data, particularly in the Mo-B-V and Mo-Si-V subsystems. As the Mo-Si-B-V alloy system is relatively novel, existing databases do not yet cover the entire compositional space with high fidelity. In the present work, the modelling was therefore focused on the Mo- and V-rich regions, which are of particular relevance for the investigated compositions. The thermodynamic parameters used were based on available literature data as well as new experimental results generated during this study. Further refinement of the database will be possible as more experimental data become available, especially for currently underexplored regions of the quaternary phase diagram.

Density measurements reveal an additional density reduction of Mo-V-Si-B alloys due to Ti doping, which is less pronounced in the V-substituted alloys. This is attributed to the fact that these alloys exhibit a lower V concentration, leading to higher densities compared to the Mo-substituted alloys. The results show that the density of these alloy systems is significantly lower than that of the Mo-9Si-8B alloy (9.4 g/cm^3^) [40] and even lower than that of commercial nickel-based superalloys like In 713C (7.95 g/cm^3^), In 100 (7.91 g/cm^3^) and CMSX-4 (8.7 g/cm^3^) [41,42].

### 3.2. Microstructure Evolution During Mechanical Alloying and Heat Treatment

**Figure 3:**
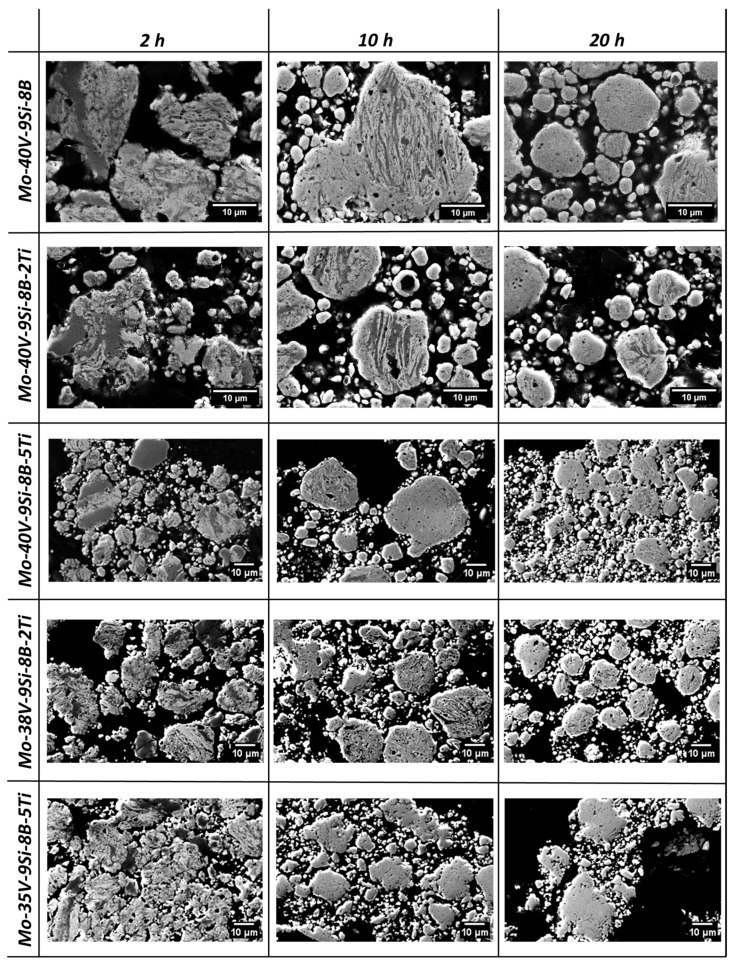
The microstructures of the mechanically alloyed powders after 2 h, 10 h and 20 h of milling. As milling time increases, a characteristic lamellar structure develops, leading to progressive microstructural homogenization.

The progress of milling can be monitored very well by analysing microstructure formation as a function of milling time. The SEM images in Figure 3 show the progress of milling in different states for all alloys. Since the microstructures revealed no significant differences after 1 h and 2 h, nor after 5 h and 10 h of milling, the microstructures after 2 h and 10 h are shown as examples, along with 20 h of milling as the maximum milling time investigated in this study. In the first hours of milling, larger light grey Mo-rich phase and dark grey V-rich phase regions can be observed within the individual powder particles. As the milling time increases up to 10 h, a lamellar structure forms, indicating that repeated comminution and cold-welding processes are the working mechanisms during mechanical alloying. In addition, V is increasingly dissolved in the (Mo,V)_ss_ phase and vice versa, and as a result, the microstructure becomes more homogeneous. However, after 20 h of milling, a completely homogeneous microstructure with only single-phase particles is not yet achieved, since lamellar structures can still be detected in some particles of all alloys, which in turn indicates that the mechanical alloying of the powders is not entirely completed at this point. Compared to previous studies on Mo-9Si-8B [8] and Mo-40V-9Si-8B [17], where a homogeneous microstructure was achieved after 20 h of milling using the higher energy PM400 mill (200 rpm), the present results indicate that the lower energy PM100 setup may not have provided sufficient energy input for full alloying.

However, when directly compared to Becker et al. [17], who used the same alloy composition (Mo-40V-9Si-8B) but without Ti and with a higher energy milling setup (PM400, 200 rpm), the present study shows that even with lower energy input, the addition of TiH_2_ enables the formation of a supersaturated solid solution and a comparable multiphase microstructure after heat treatment. Notably, the Ti-doped alloys exhibited finer particle sizes, higher microstrain, and in some cases increased microhardness already in the as-milled state, suggesting an accelerated homogenization effect—possibly due to the fragmentation-promoting nature of TiH_2_ and its interaction with oxygen.

**Figure 4:**
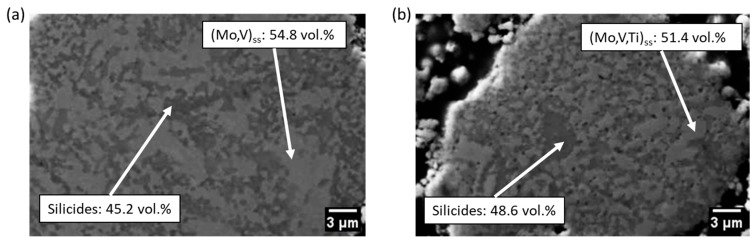
SEM images of the heat-treated (1400 °C for 1 h) powders: (**a**) Mo-40V-9Si-8B and (**b**) Mo-40V-9Si-8B-5Ti. The microstructures reveal a continuous (Mo,V)_ss_ matrix phase (light grey) with homogeneously distributed silicide particles (dark grey).

During heat treatment of the powders, the two silicide phases (Mo,V)_3_Si/(Mo,V,Ti)_3_Si and (Mo,V)_5_SiB_2_/(Mo,V,Ti)_5_SiB_2_ precipitate, which are homogeneously distributed in the continuous (Mo,V)_ss_/(Mo,V,Ti)_ss_ matrix phase, as can be seen for the two alloys Mo-40V-9Si-8 B and Mo-40V-9Si-8B-5Ti in Figure 4a,b. The microstructures are in agreement with previous studies on similarly treated Mo-9Si-8B and Mo-40V-9Si-8B alloys [6,8,17]. Although a completely homogeneous supersaturated solid solution is not evident after 20 h of mechanical alloying using the aforementioned milling parameters, the desired ultra-fine and homogenous multiphase microstructure has formed after heat treatment. Therefore, it can be concluded that the slight differences between the powder’s characteristics milled with the milling aggregates PM100 and those milled with the PM400 [6,8,17] are not significant enough to affect the microstructure after annealing.

The volume fractions of the phases were calculated using the density and mass fraction from the Rietveld refinement, with the values given in Table 2. It can be observed that in the Mo-substituted alloys Mo-40V-9Si-8B-2Ti and Mo-40V-9Si-8B-5Ti, the volume fraction of the (Mo,V)_ss_ phase decreases, while the amount of silicide phases increases with higher Ti concentrations. However, in the case of the V-substituted alloys Mo-38V-9Si-8B-2Ti and Mo-35V-9Si-8B-5Ti, the volume fractions of the (Mo,V)_ss_ phase and the silicide phases remain unchanged. In addition, the morphology and distribution of the phases are not affected by the small Ti additions.

**Figure 5:**
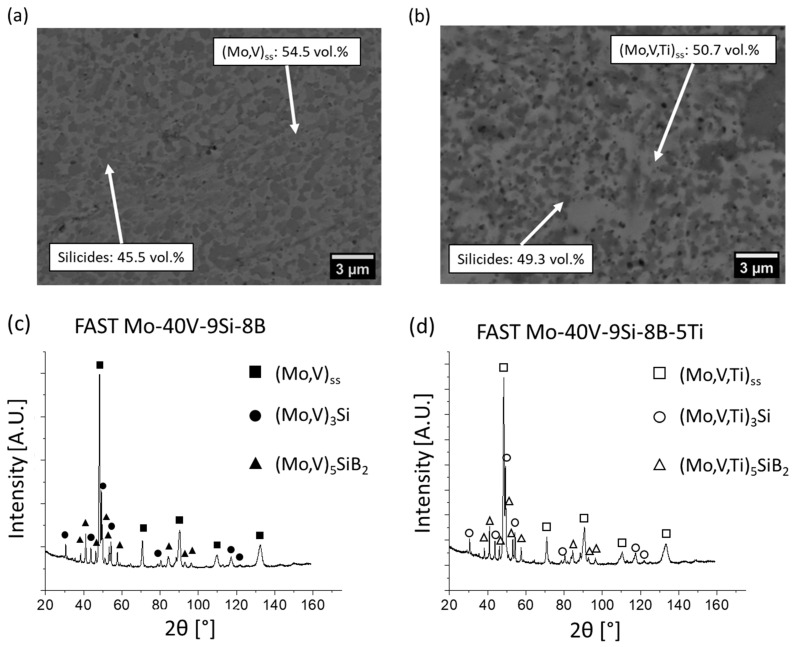
Microstructures after FAST at 1500 °C of (**a**) Mo-40V-9Si-8B and (**b**) Mo-40V-9Si-8B-5Ti, along with their corresponding X-ray diffractograms (**c**,**d**), showing the same phase distribution compared to the heat-treated powders shown in Figure 4.

It should be noted that the microstructures of the mechanically alloyed powders after heat treatment correspond well to those after sintering via the FAST process (Figure 5a,b), which is consistent with the studies of Becker et al. [17]. In addition, the volume fractions of the (Mo,V)_ss_/(Mo,V,Ti)_ss_ matrix phase and the silicide phases after heat treatment of the powders and after FAST are almost equal. Therefore, it can be concluded that the phase distribution in both the milled and heat-treated powder and the bulk material (sintered via FAST) are identical. The corresponding X-ray diffractograms in Figure 5c,d exhibit the same reflections as those of the heat-treated powders (Figure 1a,b), confirming that the microstructure consists of the same phases.

### 3.3. Particle Size Evolution During Mechanical Alloying

**Figure 6:**
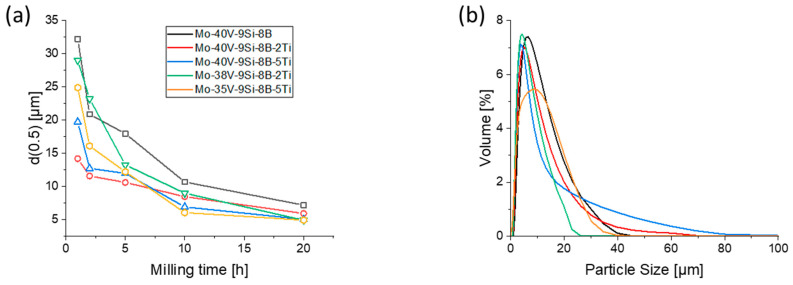
(**a**) Particle size d(0.5) as a function of milling time. The Ti-alloyed powders tend to produce smaller particles. (**b**) Particle size distribution after 20 h of milling reveals a smaller maximum and width of distribution with the addition of Ti.

Figure 6a shows the average particle size of the powders as a function of milling time for all alloys investigated. As milling time increases, the particle size d(0.5), i.e., d_50_ value of the particle size distribution, decreases due to the more pronounced comminution related to the hardening of powders by the formation of a solid solution and the progressive cold working, which corresponds to the increasing microstrain during milling (Table 1). This is due to the fact that the high impact forces of the planetary ball mill not only weld the powder particles but also effectively comminute them, as shown in previous studies [43]. The addition of small amounts of TiH_2_ seems to further reduce particle size compared to the Mo-40V-9Si-8B alloy. The TiH_2_ powder has a brittle character and is also homogeneously dispersed in the Mo powders during milling. TiH_2_ tends to fracture easily under mechanical stress, which contributes to the crushing process. During mechanical alloying, the particle size of the Ti-doped alloys decreases faster due to the easy fragmentation of the hydride [44]. All alloys exhibit a reduction in particle size in the initial stages of milling, which is consistent with Li et al. [11], who investigated Mo-12Si-8.5B nanocomposite powders treated in the planetary ball mill PM100. In the first 5 h of milling, the number of small particles increases and, in contrast to the initial powder particles, they exhibit a rough and complex shape. At this period of time, fracture and cold-welding processes occur simultaneously. After 20 h of milling, the size of the particles ranges between 4.8 and 7.2 µm, indicating a significant reduction compared to the initial powder. This suggests that steady-state conditions may have been reached, where further size reduction is minimal despite continued milling.

The particle size distribution after 20 h of milling in Figure 6b shows that with the addition of Ti, the maximum particle size distribution shifts to smaller values (except for alloy Mo-35V-9Si-8B-5Ti), which in general indicates smaller particle sizes for the Ti-alloyed powders. In addition, the width of the distribution becomes smaller with the addition of Ti (only Mo-35V-9Si-8B-5Ti shows almost the same width), which indicates that these alloys exhibit a higher fraction of small particles (smaller scattering in particle size). Therefore, it can be concluded that the addition of the hard and brittle TiH_2_ powder is an additional factor of comminution.

### 3.4. Oxygen Concentration After Mechanical Alloying and Heat Treatment

**Figure 7:**
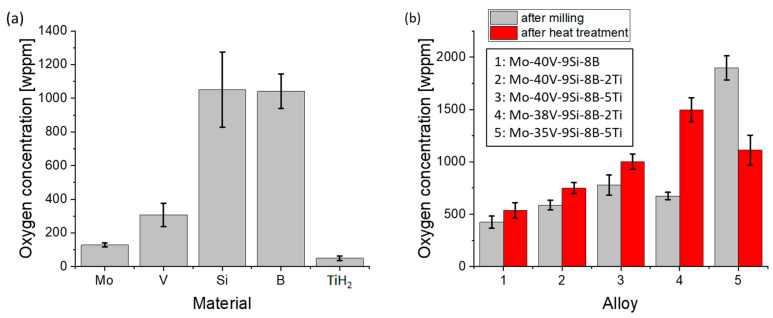
(**a**) The oxygen concentration of the elemental powders and (**b**) after high-energy ball milling and heat treatment.

The oxygen concentration of the elemental powders (Figure 7a) shows that Mo (128 ± 12 wppm, weight parts per million), V (307 ± 69 wppm) and TiH_2_ (50 ± 14 wppm) have low oxygen concentrations, while Si (1051 ± 224 wppm) and B (1042 ± 102 wppm) possess higher oxygen concentrations. As visualized in Figure 7b, the oxygen measurements show an oxygen level below 1000 wppm after 20 h for most of the alloys. Specifically, the oxygen concentration in Mo-35V-9Si-8B-5Ti after mechanical alloying was approximately twice as high compared to the other alloys. Since all alloys were processed under the same conditions, this discrepancy indicates that factors such as powder morphology, surface area or batch-to-batch variations in raw material purity influenced the initial oxygen concentration. The markedly elevated oxygen level observed in the Mo-35V-9Si-8B-5Ti alloy may be attributed to a combination of factors, including the high TiH_2_ content, which increases the specific surface area during milling due to its brittle behaviour and may locally enhance oxidation. Furthermore, partial oxidation of Ti during decomposition, possibly in the presence of surface-adsorbed moisture or residual air, could contribute to the overall oxygen uptake, even in the absence of detectable oxide phases in XRD or SEM. Although no crystalline oxide phases were detected, the presence of amorphous or nanoscale titanium oxides located on particle surfaces cannot be excluded. Due to the limitations of EDS in quantifying oxygen and the absence of a thermodynamic database including oxygen-containing phases for this quaternary system, further quantification was not attempted. Nonetheless, no signs of oxide agglomeration were observed in microstructural analysis using SEM.

It should be noted that the oxygen concentration of all alloys slightly increases during mechanical alloying, since the grinding vials are not entirely airtight, and oxygen can penetrate during milling. Consequently, the fresh surfaces, which are formed by breaking during mechanical alloying, can easily pick up residual oxygen from the process atmosphere. With the addition of Ti, the oxygen concentration increases, although the introduced oxygen of the TiH_2_ powder is negligible. A possible explanation is the oxidation of Ti to TiO_2_, which occurs even in a protective argon atmosphere, leading to the formation of an oxide layer on the TiH_2_ powder particles’ surface [45].

Figure 7b illustrates the oxygen concentration of the alloys after subsequent heat treatment of the powders. The oxygen concentration of Mo-40V-9Si-8B after heat treatment is 537 ± 74 wppm, which is less than one tenth of the value measured by Becker et al. (5787 wppm) [17] for the same alloy composition. The reason for this much smaller value is the significantly lower oxygen concentration of the as-received V powder compared with that used by Becker et al. Thus, it can be stated that the oxygen concentration of the as-received powders is crucial for the milling process.

The addition of Ti to Mo-40V-9Si-8B leads to higher oxygen concentrations (except for the Mo-35V-9Si-8B-5Ti alloy). These results are in agreement with those of Hiraoka et al. [46] who showed that the oxygen concentration in Mo-Ti alloys increases due to Ti doping. During heat treatment of the Ti-containing alloys, hydrogen is released as hydrogen gas (H_2_), especially at elevated temperatures. The hydrogen reacts with the oxygen available in these alloys to form water vapor, which is evaporated in the atmosphere. Simultaneously, Ti reacts with oxygen, leading to the formation of titanium oxides.

However, no oxides could be found in the X-ray diffractograms (Figure 1) or in the SEM images (Figure 4) for the heat-treated powders of the present studies. Nevertheless, the presence of nano-scaled or amorphous oxides below the detection limits of XRD and SEM cannot be entirely excluded. Such oxides may exist as thin films, grain boundary precipitates or finely dispersed amorphous particles. Despite this uncertainty, the available characterization data suggest that no oxides have a significant impact on the observed phase formation or overall microstructural homogeneity. The silicide phases form consistently in all samples, and no morphological anomalies or secondary oxide phases are evident.

While the oxygen concentrations in the Ti-containing alloys are slightly elevated compared to the Ti-free reference, they remain significantly lower than those reported by Becker et al. [17] or the same base composition processed without TiH_2_ (~5800 wppm). This indicates that the use of TiH_2_, despite its inherent reactivity, does not lead to excessive oxidation. On the contrary, it may even contribute to a partial deoxidizing effect during processing, potentially via hydrogen release and subsequent reaction with oxygen-containing species.

However, the oxygen concentration trend observed in Mo-35V-9Si-8B-5Ti differs significantly from that in the other investigated alloys. A possible reason for this discrepancy could be attributed to the initial oxygen concentration present after mechanical alloying. During subsequent heat treatment, the high initial oxygen concentration in Mo-35V-9Si-8B-5Ti may have influenced oxygen redistribution in the alloy. However, phase analysis and microstructural characterization of the heat-treated powder samples did not reveal the presence of volatile oxides or evidence of reduction reactions. In contrast, the alloys with a lower initial oxygen concentration exhibited an increase in oxygen concentration, which may suggest oxygen uptake from residual gas in the vacuum chamber or oxygen redistribution within the material. The measured oxygen concentration for the alloys should therefore be treated with care and seems to represent a kind of trend.

### 3.5. Microhardness Evolution During Milling and After Heat Treatment

Table 1 shows the evolution of microhardness depending on milling time. At the beginning, the microhardness strongly increases due to the different hardening mechanisms, which occur in the initial stage of mechanical alloying. Firstly, collisions between the powder and the balls lead to plastic deformation of the powder particle and consequently to a higher dislocation density (work hardening) [37]. Secondly, mechanical alloying of similar metallurgically processed Mo-Si-B powder lowers the domain size up to the lower micrometer range due to collisions between balls and between powder and balls (grain refinement) [8]. Thirdly, the addition of V and Ti, which are dissolved in the Mo lattice, further contribute to solid solution strengthening, resulting in an improvement in microhardness [18]. After 20 h, the microhardness of Mo-40V-9Si-8B has reached a value of 11 ± 3 GPa and the addition of Ti seems to increase the microhardness, except for Mo-40V-9Si-8B-2Ti (9.5 ± 2 GPa), which has a lower microhardness compared to the Ti-free alloy. On the other hand, the microhardness of Mo-35V-9Si-8B-5Ti has reached a value of 14.5 GPa but has a much smaller standard deviation (±2 GPa) compared to Mo-40V-9Si-8B. It should be noted that all microhardness measurements were conducted on individual powder particles embedded in cold-mounting resin. Due to the very small particle sizes after 20 h of milling (4.8–7.2 µm, see Figure 3 and Figure 6), it is challenging to place indentations without overlapping phase boundaries or being affected by the softer embedding material.

The results of the measured microhardness after heat treatment are shown in Table 2. For the alloys Mo-40V-9Si-8B, Mo-40V-9Si-8B-5Ti and Mo-38V-9Si-8B-2Ti, there is an increase in microhardness after heat treatment, while the microhardness in Mo-40V-9Si-8B-2Ti and Mo-35V-9Si-8B-5Ti decreases compared to the as-milled state. It should be noted that these measurements were conducted on individual powder particles embedded in cold-mounting resin.

The limited flat surface area per particle and the heterogeneous internal phase distribution further complicates precise positioning, especially when using an optical system with limited spatial resolution. These experimental constraints are compounded by the material-specific characteristics of the heat-treated powders, which exhibit an ultra-fine, multiphase microstructure with silicides ((Mo,V)_3_Si and (Mo,V)_5_SiB_2_) finely dispersed in the (Mo,V)_ss_ matrix. Based on image analysis, the average width of the silicide features is approximately 0.6 µm. Using a simplified model, the mean centre-to-centre spacing of silicide particles was estimated to be ~0.63 µm (assuming 48 vol% silicides), which is significantly smaller than the Vickers indentation size (~10–15 µm). Consequently, each indentation inevitably involves multiple phases, making it difficult to isolate phase-specific mechanical responses and contributing to increased scatter.

In addition, the powders retain a high density of lattice defects introduced during mechanical alloying, as indirectly indicated by the elevated microstrain measured via XRD (Table 1). These lattice defects (mainly point defects and dislocations) locally influence hardness and can either hinder or promote plasticity depending on their interaction with adjacent phases. Since microstrain was only measured in the as-milled state, any interpretation related to internal stresses or defect density applies solely to this condition. Potential recovery processes during subsequent heat treatment cannot be assessed based on the available data. The relatively large standard deviations in microhardness are therefore attributed to the combined effects of measurement limitations, overlapping phase contributions, lattice defects and microstructural inhomogeneities—characteristics that are typical for mechanically alloyed systems and consistent with previous observations [8,17].

It must be noted that the microhardness values given are to be understood as a medium value of the corresponding phases, i.e., a solid solution and the two silicide phases. As can be seen in Figure 4, the dispersed silicide phases are relatively small and close to each other. Due to the limited size of the silicide-free regions, it was not possible to isolate the (Mo,V)_ss_ matrix during indentation. Consequently, the standard deviations are very high, because the silicide phases (Mo,V)_3_Si/(Mo,V,Ti)_3_Si and (Mo,V)_5_SiB_2_/(Mo,V,Ti)_5_SiB_2_ exhibit a higher hardness than the (Mo,V)_ss_/(Mo,V,Ti)_ss_ matrix phase. In this context, Choe et al. [47] investigated the microhardness of Mo-12Si-8.5B and was able to show that the microhardness of Mo_5_SiB_2_ (18.5 GPa) is slightly higher compared to that of Mo_3_Si (15 GPa), which are both significantly higher than that of the Mo_ss_ matrix phase (7 GPa). The addition of 2 at. % Ti to Mo-V-Si-B seems not to affect the microhardness significantly (in the case of Mo-40V-9Si-8B-2Ti, it is even significantly lower), while 5 at. % Ti leads to a noticeable improvement in microhardness. These results are in agreement with those of Becker et al. [18], who showed that the addition of 5 at. % Ti is sufficient to increase the microhardness of pure Mo due to solid solution hardening. However, in the case of Mo-35V-9Si-8B-5Ti, this increase in microhardness is not as pronounced compared to Mo-40V-9Si-8B-5Ti. Therefore, the addition of 5 at. % Ti to Mo-40V-9Si-8B has the highest impact on microhardness in the present study.

## 4. Conclusions

The present study investigated the effects of small titanium additions (2 and 5 at. %), which were added as TiH_2_ powders, on the microstructure, phase evolution, oxygen concentration and microhardness of mechanically alloyed Mo-40V-9Si-8B alloys.

All compositions formed a supersaturated (Mo,V,Ti) solid solution during mechanical alloying, which decomposed into a stable three-phase microstructure consisting of (Mo,V)_ss_, (Mo,V)_3_Si and (Mo,V)_5_SiB_2_ or (Mo,V,Ti)_ss_, (Mo,V,Ti)_3_Si and (Mo,V,Ti)_5_SiB_2_ after heat treatment. Compared to the Ti-free alloy, the Ti-containing variants showed slightly finer particle sizes, higher microstrain and, in the case of 5 at. % Ti, a modest increase in microhardness. Although the Ti-doped alloys exhibited slightly elevated oxygen concentrations compared to the Ti-free composition, the overall values remained significantly lower than those reported in earlier studies using conventional powder routes—demonstrating the potential of TiH_2_ as a reactive alloying agent and deoxidizing precursor.

The brittleness of TiH_2_ affects powder refinement during milling, while its decomposition may support a more homogeneous element distribution. Although the overall oxygen content in the Ti-containing alloys was slightly higher than in the Ti-free reference, it remained significantly lower than in comparable studies using elemental powders—indicating that TiH_2_ does not critically promote oxygen uptake and may still offer benefits in oxygen-sensitive processing environments.

Based on the promising results of this study, future research should focus on evaluating the high-temperature performance of these alloys, including their mechanical behaviour in a broad temperature regime (including flexural and tensile deformation, creep and fracture behaviour), as well as their environmental effects (oxidation, corrosion). With their reduced density and complex multiphase architecture, these Ti- and V-modified Mo–Si–B alloys are promising candidates for high-temperature structural applications such as turbine components or thermal barrier systems.

## Figures and Tables

**Table 1:** The XRD results as a function of milling time for the five alloys investigated.

Alloy	Mo-40V-9Si-8B			Mo-40V-9Si-8B-2Ti			Mo-40V-9Si-8B-5Ti			Mo-38V-9Si-8B-2Ti			Mo-35V-9Si-8B-5Ti		
Milling time [h]	***2***	***10***	***20***	***2***	***10***	***20***	***2***	***10***	***20***	***2***	***10***	***20***	***2***	***10***	***20***
Lattice parameter Mo(V)_ss_ [Å]	3.146	3.105	3.087	3.141	3.119	3.100	3.139	3.111	3.106	3.140	3.107	3.105	3.143	3.106	3.090
Lattice parameter V(Mo)_ss_ [Å]	3.034	3.050	3.079	3.040	3.059	3.085	3.043	3.066	3.069	3.040	3.062	3.072	3.045	3.078	3.072
Microstrain Mo(V)_ss_ [10^−3^]	5.3	8.2	8.7	4.8	6.8	8.9	5.0	6.1	5.3	4.9	4.9	4.9	4.7	7.3	8.1
Microstrain V(Mo)_ss_ [10^−3^]	3.4	6.5	13.2	3.2	7.2	10.0	3.4	9.2	7.2	3.5	6.7	7.1	3.9	10.9	8.1
Phase fraction Mo [wt.%]	45.1	12.1	10.1	62.5	22.9	12.0	67.5	33.2	19.5	64.4	35.1	18.8	68.6	15.0	7.9
Phase fraction Mo(V)_ss_ [wt.%]	21.2	55.5	44.3	6.7	32.3	20.1	3.7	26.5	25.8	6.7	20.1	19.0	6.5	32.2	22.2
Phase fraction V(Mo)_ss_ [wt.%]	33.7	32.4	45.6	30.8	44.9	68.0	28.8	40.3	54.7	28.9	44.8	62.2	24.8	52.8	70.0
Concentration of V in Mo(V)_ss_ [wt.%]	2.2	34.4	48.4	4.3	22.6	47.6	6.0	29.4	33.3	5.4	32.8	34.3	3.2	33.8	46.4
Concentration of Mo in V(Mo)_ss_ [wt.%]	7.4	20.8	17.0	11.9	27.5	43.8	14.4	33.7	36.3	12.2	30.3	38.4	15.9	43.8	38.3
Microhardness [GPa]	8 (±3)	10.5 (±2)	11 (±3)	6.5 (±2)	8 (±2)	9.5 (±2)	5 (±1)	10.5 (±3)	11.5 (±2)	8 (±1)	11 (±2)	11.5 (±2)	7.5 (±1)	13 (±2)	14.5 (±2)

**Table 2:** XRD parameters including lattice parameter, phase concentration and results of microhardness measurements after heat treatment at 1400 °C.

Alloy	Mo-40V-9Si-8B	Mo-40V-9Si-8B-2Ti	Mo-40V-9Si-8B-5Ti	Mo-38V-9Si-8B-2Ti	Mo-35V-9Si-8B-5Ti
Lattice parameter (Mo,V)_ss_/(Mo,V,Ti)_ss_ [Å]	3.090	3.089	3.084	3.098	3.083
Lattice parameter (Mo,V)_3_Si/(Mo,V,Ti)_3_Si [Å]	4.798	4.796	4.796	4.799	4.800
Lattice parameter (Mo,V)_5_SiB_2_/(Mo,V,Ti)_5_SiB_2_ [Å]	5.867 10.883	5.865 10.876	5.876 10.915	5.870 10.885	5.890 10.935
Phase fraction (Mo,V)_ss_/(Mo,V,Ti)_ss_ [wt.%] BCC	59.0	56.8	54.7	58.7	54.8
Phase fraction (Mo,V)_3_Si/(Mo,V,Ti)_3_Si [wt.%] A15	17.5	19.1	21.0	18.3	21.9
Phase fraction (Mo,V)_5_SiB_2_/(Mo,V,Ti)_5_SiB_2_ [wt.%] T2	23.5	24.1	24.3	23.0	23.3
Microhardness [GPa]	12 (±3)	9 (±2)	13 (±2)	12 (±2)	12 (±2)
Theoretical density [g/cm^3^]	7.91	7.78	7.79	7.88	7.82
Measured density [g/cm^3^]	7.88 *		7.54 *		

* Measured after field-assisted sintering (FAST) of the mechanically alloyed powders.

## Data Availability

The original contributions presented in this study are included in the article. Further inquiries can be directed to the corresponding author.
